# *Papaver nudicaule* (Iceland poppy) alleviates lipopolysaccharide-induced inflammation through inactivating NF-κB and STAT3

**DOI:** 10.1186/s12906-019-2497-5

**Published:** 2019-04-29

**Authors:** Jae-Hyeon Oh, Miyong Yun, Dain Park, In Jin Ha, Chang-Kug Kim, Do-Wan Kim, Eun-Ok Kim, Seok-Geun Lee

**Affiliations:** 1 Genomics Division, Department of Agricultural Biotechnology, National Institute of Agricultural Sciences Rural Development Administration, Jellabuk-do, 54874 Republic of Korea; 2 Bioindustry & Bioresource Engineering, College of Life Sciences, Sejong University, 209 Neungdong-ro, Gwangjin-gu, Seoul, 05006 Republic of Korea; 3 Department of Science in Korean Medicine, Kyung Hee University, Seoul, 02447 Republic of Korea; 4 Korean Medicine Clinical Trial Center, Korean Medicine Hospital, Kyung Hee University, Seoul, 02447 Republic of Korea; 5 KHU-KIST Department of Converging Science & Technology, and Bionanocomposite Research Center, Kyung Hee University, Seoul, 02447 Republic of Korea

**Keywords:** *Papaver nudicaule*, Inflammation, Macrophage, NF-κB, STAT3

## Abstract

**Background:**

*Papaver nudicaule* belongs to the *Papaveraceae* family, which is planted as an annual herbaceous species generally for ornamental purpose. *Papaver rhoeas* in the same family has been reported to have various pharmacological activities such as antioxidant and analgesic effects. In contrast, little is known about the pharmacological activity of *Papaver nudicaule*. In this study, the anti-inflammatory activity of *Papaver nudicaule* extracts and the action mechanisms were investigated in RAW264.7 macrophage cells.

**Methods:**

To investigate the anti-inflammatory activity of five cultivars of *Papaver nudicaule* with different flower color, samples were collected from their aerial parts at two growth stages (60 and 90 days) and their ethanol extracts were evaluated in the lipopolysaccharide (LPS)-treated RAW264.7 cells by measuring nitric oxide (NO) and prostaglandin E2 (PGE2) levels. Interleukin 1-beta (IL-1β), Interleukin-6 (IL-6) and Tumor necrosis factor alpha (TNF-α) production were also analyzed by RT-PCR and multiplex assays. Nuclear Factor-kappa-light-chain-enhancer of activated B cells (NF-κB) and Signal transducer and activator of transcription 3 (STAT3) signaling pathways were examined using western blotting and luciferase reporter assays to reveal the action mechanism of *Papaver nudicaule* extracts in their anti-inflammatory activity.

**Results:**

All of the *Papaver nudicaule* extracts were effective in reducing the LPS-induced NO, which is an important inflammatory mediator, and the extract of *Papaver nudicaule* with white flower collected at 90 days (NW90) was selected for further experiments because of the best effect on reducing the LPS-induced NO as well as no toxicity. NW90 lowered the LPS-induced PGE2 level and decreased the LPS-induced Nitric oxide synthase 2 (NOS2) and Cyclooxygenase 2 (COX2). In addition, NW90 reduced the LPS-induced inflammatory cytokines, IL-1β and IL-6. Furthermore, NW90 inhibited the LPS-induced activation of NF-κB and STAT3.

**Conclusions:**

These results indicate that NW90 may restrain inflammation by inhibiting NF-κB and STAT3, suggesting the potential therapeutic properties of *Papaver nudicaule* against inflammatory disease.

**Electronic supplementary material:**

The online version of this article (10.1186/s12906-019-2497-5) contains supplementary material, which is available to authorized users.

## Background

Acute inflammation is one of the defensive reactions of biological tissues against external stimuli and is a protective response involving immune cells and molecular mediators. However, hyperinflammatory responses can cause severe sepsis, resulting in multiple organ failure as well as high mortality [1–3]. Therefore, anti-inflammation is an important issue in controlling various diseases. The macrophage plays an important role in the biological defense in the early stage of inflammation by producing inflammatory mediators, including cytokines. Several inflammatory mediators such as NO, PGE2, and cytokines (IL-6, IL-1β, and TNF-α) have been reported to play key roles in the inflammatory response [4]. For this purpose, inhibiting macrophage function inclusive of inflammatory mediators has potential as a therapeutic agent in the treatment of various inflammatory diseases [5–7].

*Papaver nudicaule* (Iceland poppy, Family: *Papaveraceae*) is a short-lived perennial plant which has been cultivated mainly for ornamental purpose in many countries including Asia and Europe [8]. As a matter of fact, five cultivars of *Papaver nudicaule* blooms have been identified (yellow, orange, pink, scarlet and white). The cultivar of white color is the dominant one, while the others are recessive [9]. In Tibet, Europe, and North Asia, the flowers and seeds have been used as mild diaphoretic by folk medicines [10, 11] and the leaves have been used as a source of vitamin C [12]. Despite the existence of these folk remedies, their pharmacological activity and action mechanism has not been revealed yet.

In this study, we investigated the inhibitory effects of ethanol extracts of the five cultivars of *Papaver nudicaule* on the lipopolysaccharide (LPS)-induced inflammation in RAW264.7 cells and its mechanism.

## Materials and methods

### Preparation of *Papaver nudicaule* extracts

The aerial parts of *Papaver nudicaule* harvested at two different growth stages (60 and 90 days) were provided by the National Institute of Agricultural Science, Rural Development Administration (Republic of Korea). All five cultivars of *Papaver nudicaule* with different flower colors were used as noticed in Table 1. Every voucher specimen was identified by Dr. Do-Wan Kim in the Genomics Division of the National Institute of Agricultural Science [13]. Here we abbreviate the extract of *Papaver nudicaule* with white flower harvested at 60 days after seeding to NW60 and the same way for the other extracts (Table 1). The specimens used in this study were deposited in the Genomics Division of the National Institute of Agricultural Science (Republic of Korea). Aerial parts of *Papaver nudicaule* were lyophilized and then ground into a fine powder. The ethanol extraction methods are previously described [14]. 2 g of each sample was ultrasonicated for 30 mins with 5 ml of ethanol, and then centrifugation was performed for 15 mins at 13,000 rpm at 4 °C. The supernatants were filtered through the 0.2 μm polytetrafluoroethylene syringe filter (Thermo Scientific, Waltham, MA). Each solvent was then evaporated using speed vacuum (Thermo/Savant SPC VAC 2010, Waltham, MA). Each sample extracted was dissolved in DMSO at a concentration of 500 mg/ml based on the weight of the ground powder after initial lyophilization and stored at − 70 °C until used in the experiments.

**Table 1:** Five kinds of *Papaver nudicaule* with different flower color and the abbreviation

Species	Color	Abbreviation
*Papaver nudicaule*	White	NW
*Papaver nudicaule*	Orange	NO
*Papaver nudicaule*	Yellow	NY
*Papaver nudicaule*	Scarlet	NS
*Papaver nudicaule*	Pink	NP

### Cell culture and reagents

RAW264.7 murine macrophage cells were maintained in Dulbecco’s modified Eagle’s medium (DMEM) containing 10% FBS and 1% antibiotic/antimycotic in a humidified incubator with 5% CO_2_ at 37 °C. LPS (*Escherichia coli* serotype 0111: B4) and 3-(4, 5-dimethylthiazol-2-yl)-2, 5-diphenyl-tetrazolium bromide (MTT) were purchased from Sigma-Aldrich (St. Louis, MO, USA). A Nitric oxide detection kit was purchased from iNtRON Biotechnology (Sungnam, Republic of Korea).

### Cell viability assays

Cell viability was measured by MTT assays. Cells (1 × 10^4^ cells/well) were seeded in 96-well plates and incubated overnight. Cells were treated with LPS and *Papaver nudicaule* extracts for 24 h [15], and then 20 μl of 2 mg/ml MTT solution was added to each well. The cells were incubated at 37 °C for 3 h. Formazan crystals were dissolved by DMSO and resulting absorbance value was measured using microplate reader (Molecular Devices, California, USA) at 570 nm. Data are presented as mean ± standard deviation (SD) from at least three independent experiments in triplicate.

### Nitric oxide assays

The nitrite concentration in the culture supernatant was analyzed as an indicator of NO production using Griess reagent. RAW264.7 cells (5 × 10^5^ cells/well) were seeded in 6-well plates and incubated overnight. Cells were pretreated with *Papaver nudicaule* extracts 1 h prior to LPS (100 ng/ml) treatment for 24 h [15]. Then the culture supernatants were mixed with Griess reagent [equal volumes of 1% (*w*/*v*) sulfanilamide in 5% (*v*/v) phosphoric acid and 0.1% (w/v) naphtylethylenediamine-HCL], incubated at room temperature for 10 min, and the absorbance at 540 nm was measured using a microplate reader. Data are presented as mean ± SD from at least three independent experiments in triplicate.

### Real-time quantitative PCR

Cells (5 × 10^5^ cells/well) were seeded in 6-well plates and incubated overnight. Then the cells were pretreated with *Papaver nudicaule* extracts 1 h prior to LPS (100 ng/ml) treatment for 24 h. Total RNA was isolated using TRI Reagent solution (Ambion, Waltham, MA, USA) according to the manufacturer’s instructions. Total RNA (1 μg) isolated from cells was reverse transcribed to cDNA using PrimeScript first-strand cDNA synthesis kit (Takara Korea Biomedical Inc., Seoul, Republic of Korea) according to the manufacturer’s instructions. Amplification of each cDNA was monitored using Sensi FAST SYBR No-ROX kit (Bioline, Taunton, MA, USA) on a StepOnePlus instrument (Waltham, Massachusetts, USA). Specific primers used as following: IL-6 (forward: 5′-CCACGGCCTTCCCTACTTC-3′, and reverse: 5′-TTGGGAGTGGTATCCTCTGTGA-3′), TNF-α (forward: 5′-CACCGTCAGCCGATTTGC-3′, and reverse: 5′-TTGACGGCAGAGAGGAGGTT-3′), IL-1β (forward: 5′-AGTTGACGGACCCCAAAAGAT-3′, and reverse: 5′-GGACAGCCCAGGTCAAAGG-3′), GAPDH (forward: 5′-CAAGGCTGTGGGCAAGGT-3′, and reverse: 5′-GGAAGGCCATGCCAGTGA-3′). GAPDH was used as an internal control. Data are presented as mean ± SD from at least three independent experiments in triplicate.

### Western blotting analysis

Whole cell lysates were prepared and western blotting was performed as described [16]. Cells (3 × 10^6^ cells) were seeded in 100 mm dishes and incubated overnight. The cells were pre-incubated with *Papaver nudicaule* extracts for 1 h and then incubated with LPS (100 ng/ml) and the extracts for 24 h. Primary antibodies against COX2, NF-κB p65, phospho-NF-κB p65, IκBα, phospho-IκBα, STAT3, and phospho-STAT3 were purchased from Cell Signaling Technology (Danvers, MA, USA), NOS2 from Santa Cruz Biotechnology (Santa Cruz, CA, USA), and β-actin from Sigma-Aldrich. Secondary antibodies HRP-conjugated anti-mouse IgG and anti-rabbit (1:5000–1:10000; Jackson Immuno Research Laboratories, Inc., PA, USA) were used for western blotting. Densitometric analysis of each protein band was calculated using the ImageJ software (https://imagej.nih.gov/ij/). Data are presented as mean ± SD from at least three independent experiments.

### Multiplex cytokine assays

Cells (5 × 10^5^ cells/well) were pre-incubated with *Papaver nudicaule* extracts for 1 h in 6-well plates, incubated with LPS (100 ng/ml) and the extracts for 24 h. IL-1β, IL-6, and TNF-α were measured using a Magnetic Luminex Assay kit (R&D Systems, MN, USA) and Luminex 200 array system after collecting the culture medium. Standard curves for each cytokine were generated using the kit-supplied reference cytokines. Data are presented as mean ± SD from at least three independent experiments in triplicate.

### ELISA assays

Enzyme-linked immunosorbent assay (ELISA) kits were purchased from R&D system (Minneapolis, MN, USA) for IL-1β and Biochem Inc. (Farmingdale, NY, USA) for PGE2. To measure IL-1β and PGE2 secretion in the culture supernatants, cells (5 × 10^5^ cells/well) were pre-incubated with *Papaver nudicaule* extracts for 1 h in 6 well plates and incubated with LPS (100 ng/ml) and extracts for 24 h. The culture supernatants were collected and the concentration of PGE2 and IL-1β was measured using a microplate reader. Data are presented as mean ± SD from at least three independent experiments in triplicate.

### Transient transfection and luciferase reporter assays

NF-κB-Luc and pSTAT3-Luc vectors were previously described [16, 17]. RAW264.7 cells were transiently transfected with NF-κB-Luc or pSTAT3-Luc using Lipofectamine 2000 (Invitrogen, Carlsbad, CA). Transfection efficiency (> 24% for NF-κB-Luc and > 34% pSTAT3-Luc) was confirmed by measuring the bioluminescence intensity with flow cytometry (FACS Canto II, BD Biosciences, San Jose, CA) [18]. One day after transfection, the cells were re-plated into 24 well plates (5 × 10^4^ cells/well) and incubated overnight. Then the cells were pre-treated with *Papaver nudicaule* extracts 1 h prior to LPS (100 ng/ml) treatment. Following incubation for 3 or 24 h, luciferase assays were performed using a luciferase assay system (Promega, Madison, WI, USA) according to the manufacturer’s instructions. Data are presented as mean ± SD from at least three independent experiments in triplicate.

### Statistical analyses

Statistical analysis was performed with the SigmaPlot 10.0 software (Systat Software, Inc., San Jose, CA, USA). An unpaired Student’s *t*-test was performed between mock and LPS treatment or LPS alone and co-treatment of LPS with *Papaver nudicaule* extract to calculate the *P*-value [19]. *P* < 0.05 was considered significant.

## Results

### Extracts of *Papaver nudicaule* decrease the LPS-induced NO and PGE2

**Fig. 1:**
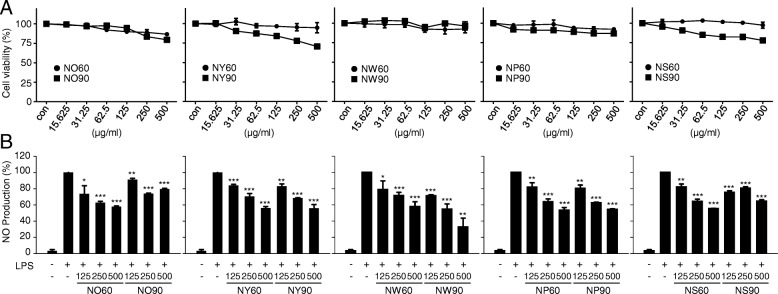
Effects of *Papaver nudicaule* extracts on cytotoxicity and NO production. **a** RAW264.7 cells were treated with *Papaver nudicaule* extracts (15.625–500 μg/ml) for 24 h. Cell viability was measured by MTT assays. **b** RAW264.7 cells were treated with *Papaver nudicaule* extracts (125–500 μg/ml) and LPS (100 ng/ml) for 24 h. NO production was examined in the culture supernatants. Data are presented as mean ± SD from at least three independent experiments in triplicate. *P < 0.05, **P < 0.01 and ***P < 0.001 indicate statistically significant differences compared with the group treated with LPS alone

In order to investigate the potential anti-inflammatory effect of *Papaver nudicaule*, we first examined the cytotoxicity of all five cultivars of *Papaver nudicaule* in RAW264.7 cells. Analysis of cell viability after treatment of each extract did not show any significant cytotoxicity up to 500 μg/ml even though NO90, NY90, and NS90 were slightly cytotoxic at higher concentrations (Fig. 1a). The IC_50_ values of all five cultivars of *Papaver nudicaule* extracts were over 2000 μg/ml, indicating mild or no toxicity of *Papaver nudicaule* cultivars we used (Additional file 1: Table S1). To examine the anti-inflammatory effects of *Papaver nudicaule* extracts, we then evaluated NO production in RAW264.7 cells treated with LPS in the presence or absence of each extract. As shown in Fig. 1b, LPS dramatically increased NO and all of the extracts decreased the LPS-induced NO production. Moreover, their inhibitory effects on NO production were in a dose-dependent manner except NO90 and NS90. In particular, only NW90 noticed that its IC_50_ value on the inhibitory effect on the LPS-induced NO production was less than 500 μg/ml. Based on these results indicating no toxicity and the most effective in decreasing the LPS-induced NO production, NW90, the extract of *Papaver nudicaule* with white flower harvested at a cultivation period of 90 days, was selected and used for further experiments.

**Fig. 2:**
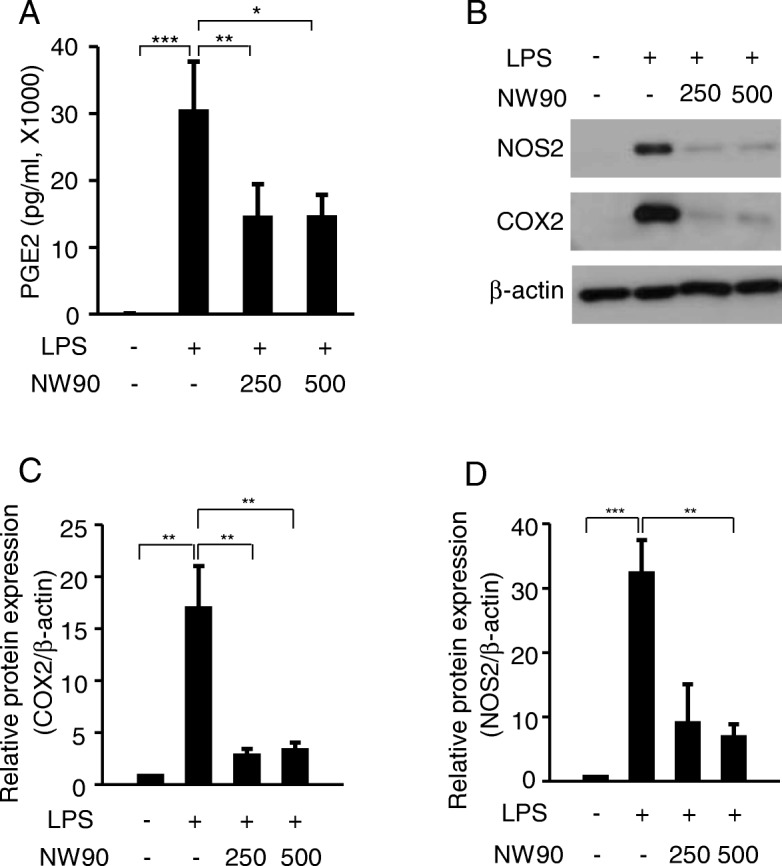
Effects of *Papaver nudicaule* extract NW90 on the expression of PGE2, COX2, and NOS2. RAW264.7 cells were treated with NW90 extract (250 and 500 μg/ml) and LPS (100 ng/ml) for 24 h. **a** The PGE2 production was measured in the culture supernatants. **b** Cells were lysed and the expression of COX2 and NOS2 was examined by western blotting. β-actin was used as an internal control. **c**, **d** Quantification of COX2 and NOS2 expression with normalization to the β-actin protein levels were analyzed by densitometry analysis of every band using the ImageJ software. Data are presented as mean ± SD from at least three independent experiments. *P < 0.05, **P < 0.01 and ***P < 0.001 indicate statistically significant differences compared with the group treated with LPS alone

To further verify the effect of NW90 on the LPS-induced inflammatory response, we examined its effect on the expression of PGE2 in RAW264.7 cells. As shown in Fig. 2a, LPS greatly increased PGE2 production and NW90 significantly decreased the LPS-induced PGE2. To understand the molecular mechanism underlying the inhibition of LPS-induced NO and PGE2 production by NW90, we evaluated the expression levels of NOS2 and COX2, which are key enzymes to regulate NO and PGE2 production, respectively [20–22]. As shown in Fig. 2b-d, NW90 inhibited the LPS-induced expression of NOS2 and COX2. These results indicated that the extract of the *Papaver nudicaule* cultivar with white flower NW90 decreases the LPS-induced NO and PGE2 by reducing the expression of NOS2 and COX2, respectively.

### Extract of NW90 reduces the LPS-induced inflammatory cytokines

**Fig. 3:**
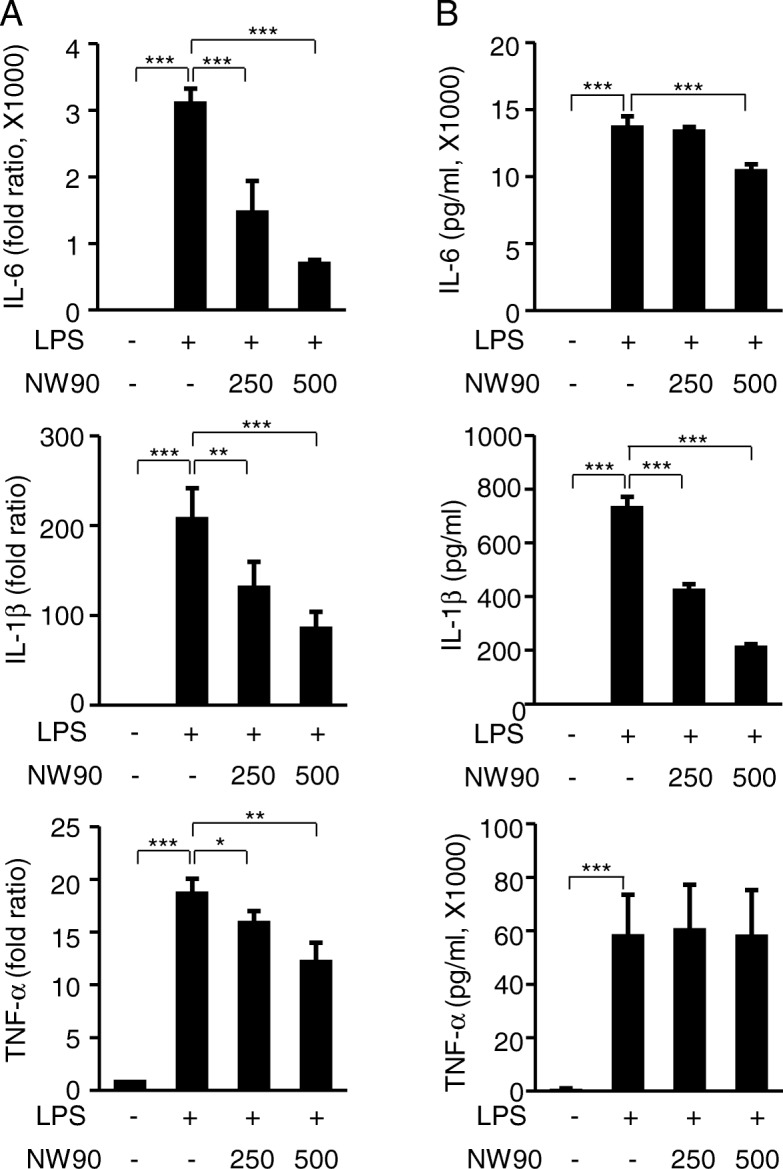
Effects of *Papaver nudicaule* extract NW90 on the production of inflammatory cytokines. RAW264.7 cells were treated with NW90 (250 and 500 μg/ml) and LPS (100 ng/ml) for 24 h. **a** Expression levels of IL-6, IL-1β, and TNF-α mRNA were measured by real-time quantitative PCR. GAPDH was used as an internal control. **b** Secretion levels of IL-6, IL-1β and TNF-α were determined by multiplex assays using the culture supernatants. Data are presented as mean ± SD from at least three independent experiments in triplicate. *P < 0.05, **P < 0.01 and ***P < 0.001 indicate statistically significant differences compared with the group treated with LPS alone

We then analyzed the effect of NW90 on the expression and secretion of inflammatory cytokines such as IL-1β, IL-6, and TNF-α, which are potent activators of inflammatory responses in macrophage [23]. LPS highly increased mRNA expression and secretion of IL-1β, IL-6, and TNF-α (Fig. 3). In contrast, NW90 significantly decreased mRNA expression levels of the LPS-induced IL-1β, IL-6, and TNF-α (Fig. 3a). In addition, NW90 reduced the secretion of the LPS-induced IL-1β and IL-6, but no inhibitory effect on the secretion of TNF-α (Fig. 3b). These results suggested that NW90 may have anti-inflammatory effects by reducing the LPS-induced IL-1β and IL-6 production and secretion.

### Extract of NW90 inhibits the LPS-induced NF-κB and STAT3 activation

**Fig. 4:**
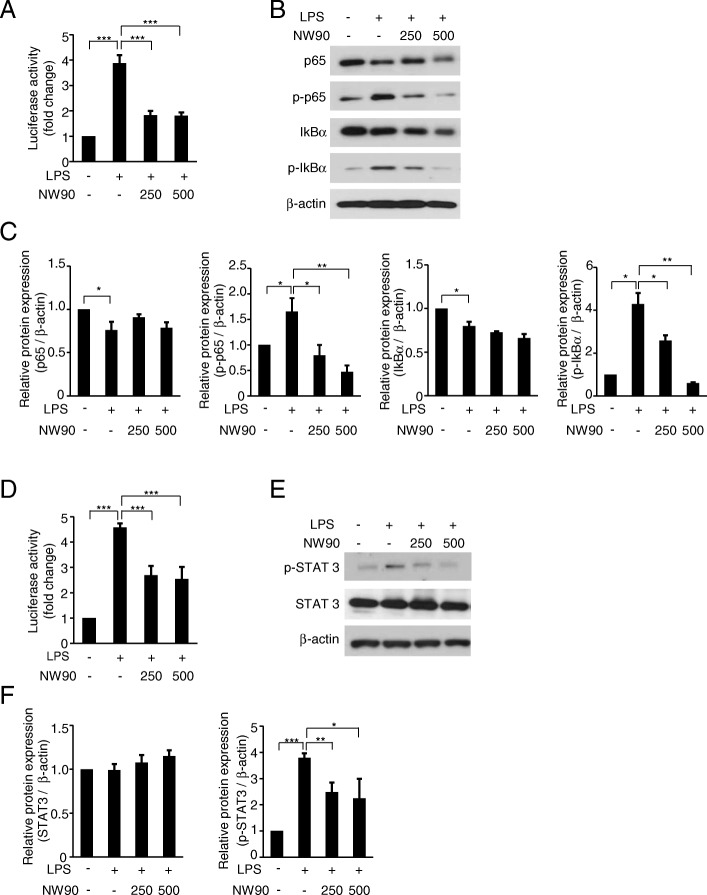
Effects of *Papaver nudicaule* extract NW90 on the NF-κB and STAT3 signaling pathways. **a**, **d** RAW264.7 cells transfected with the NF-κB-Luc or pSTAT3-Luc were treated with NW90 extract (250 and 500 μg/ml) and LPS (100 ng/ml). Luciferase activities were measured in cell lysates at 3 (**a**) or 24 (**d**) hours after the treatment. Data are presented as mean ± SD from at least three independent experiments in triplicate. **b**, **e** Expression and phosphorylation of p65, IκBα and STAT3 were analyzed by western blotting in the treated RAW264.7 cells as indicated. β-actin was used as an internal control. **c**, **f** Quantification of p65, p-p65, IκBα, p-IκBα, STAT3, and p-STAT3 expression with normalization to the β-actin protein levels were analyzed by densitometry analysis of every band using the ImageJ software. Data are presented as mean ± SD from at least three independent experiments. *P < 0.05, **P < 0.01 and ***P < 0.001 indicate statistically significant differences compared with the group treated with LPS alone

Since transcription factors NF-κB and STAT3 are known to regulate the expression of inflammatory mediators such as NOS2, COX2 and inflammatory cytokines [24, 25], we next investigated the effect of NW90 on the LPS-induced transcriptional activation of NF-κB and STAT3. As shown in Fig. 4a and d, LPS induced transcriptional activity of both transcription factors, and NW90 significantly decreased the LPS-induced transcriptional activation of NF-κB and STAT3. Accordingly, NW90 reduced the LPS-induced phosphorylation levels of IκBα, p65 (Fig. 4b and c) and STAT3 (Fig. 4e and f), indicating NW90-mediated inactivation of NF-κB and STAT3 signaling pathways. These results suggested that NW90 extract is a potent inhibitor of NF-κB and STAT3.

## Discussion

In this study, the anti-inflammatory activities of *Papaver nudicaule* against LPS-induced inflammation in RAW264.7 cells were examined. NW90 inhibited the LPS-induced production of NO and PGE2 by regulating the expression of NOS2 and COX2 and also suppressed the LPS-induced production of inflammatory cytokines, IL-1β and IL-6. We further confirmed that NW90 inactivated the LPS-induced NF-κB and STAT3 activation. These results indicate that *Papaver nudicaule* is an herbal plant with anti-inflammatory activity through inactivating NF-κB and STAT3, suggesting a potential to be developed as an anti-inflammatory agent. NF-κB is a major downstream transcription factor regulating the expression of inflammation-related genes during the induction of inflammatory stimuli such as LPS [26, 27] and is also associated with many chronic inflammatory diseases such as inflammatory bowel disease, atopic dermatitis and rheumatoid arthritis [28]. Therefore, the anti-inflammatory activity of *Papaver nudicaule* through NF-κB regulation is likely to be applied to chronic inflammatory disease. In addition, STAT3 is another important transcription factor involved in the immune response and inflammation and is known to collaborate with NF-κB to control inflammation [27]. Many inflammatory cytokines induced by NF-κB or STAT3 can positively feedback to activate STAT3 and NF-κB [29]. IL-6 induced by NF-κB activates STAT3, resulting in the increased expression of STAT3-target genes [30]. Therefore, dual inhibition of NF-κB and STAT3 is an attractive therapeutic strategy for treating inflammatory diseases and we in the present study suggest *Papaver nudicaule* as a novel candidate for developing a new anti-inflammatory drug targeting both NF-κB and STAT3.

Studies on the development of anti-inflammatory drug and dietary food using herbal plants as complementary medicine and using phytochemicals have been going for a long time [31]. Various phytochemicals such as flavonoids and alkaloids have been isolated from various plants and their biological activities have been confirmed [32, 33]. Chemical compounds and their pharmacological activities of other species in *Papaver* such as *Papaver somniferum* and *Papaver rhoeas* have been characterized well [14, 34]. In contrast, few studies about *Papaver nudicaule* have been done. Any pharmacological or biological activity of *Papaver nudicaule* has not been uncovered yet, and just a few alkaloids including chelidonine and allocryptopine have been known in *Papaver nudicaule* [18, 35, 36]. Although chelidonine is a minor alkaloid in *Papaver nudicaule*, recent studies indicated that chelidonine extracted from *Chelidonium majus* suppresses the TNF-α and LPS-induced inflammation by inhibiting NF-κB in HCT 116 and RAW264.7 cells, respectively [37, 38]. We, thus, analyzed the two known compounds chelidonine and allocryptopine in *Papaver nudicaule* by LC-MS analysis. Allocryptopine was detected in every extract of all five cultivars of *Papaver nudicaule* including NW90 (Additional file 2: Figure S1). However, chelidonine was not observed in all our samples (data not shown). It is considered that this type of difference is generally caused by various reasons such as usage of another part of the plant (leaf, flower, stem, root, etc.), environmental issues for cultivation, and the cultivation period. We used *Papaver nudicaule* cultivated in the Republic of Korea, but the other group collected it from Mongolia [18, 36].

In the present study we notice that *Papaver nudicaule* alleviates the LPS-induced inflammation by suppressing NOS2, COX2 and inflammatory cytokines IL-1β and IL-6 through inhibiting NF-κB and STAT3 pathways, indicating a potential pharmacological activity of *Papaver nudicaule* for the first time. In contrast to the other *Papaver* species, *Papaver nudicaule* has not received any attention in the field of pharmacology or herbal medicine. Beginning our first presentation, further studies about *Papaver nudicaule* in the context of other pharmacological and biological activities and pharmaco-chemical analysis will warranty that *Papaver nudicaule* could be a useful herbal plant for human beings, not just an ornamental poppy.

## Conclusion

Our experimental results indicate that *Papaver nudicaule* extracts have the anti-inflammatory activity to decrease the inflammatory response induced by LPS through inhibiting NF-κB and STAT3 pathways. Our findings suggest that *Papaver nudicaule* could be a potentially useful herbal plant with an anti-inflammatory effect.

## Additional files

Additional file 1: **Table S1.** IC_50_ values of *Papaver nudicaule* cultivars. (DOCX 14 kb)

Additional file 2: **Figure S1.** Extracted ion chromatograms of (A) authentic standard of allocryptopine and (B) blank solution and allocryptopine from samples analyzed by LC-QTOF in the ethanol extracts of aerial parts of (C) NW (D) NO (E) NY (F) NS, and (G) NP at a cultivation period of 90 days. **Supplementary Methods.** The liquid chromatography-mass spectrometry system consisted of a Thermo Scientific Vanquish UHPLC system (Thermo Fisher Scientific, Sunnyvale, CA, USA) with an Acquity UPLC HSS T3 column (2.1 mm × 100 mm, 1.7 μm; Waters) and a Triple TOF 5600^+^ mass spectrometer system (Triple TOF MS; QTOF, Sciex, Foster City, CA, USA). Data acquisition and processing were carried out using Analyst TF 1.7, PeakVeiw 2.2 and MasterView software (Sciex, Foster City, CA, USA). (ZIP 185 kb)

## Abbreviations

(IL-1β): Interleukin 1 beta
(IL-6): Interleukin-6
COX2: Cyclooxygenase 2
DMSO: Dimethyl sulfoxide
IC_50_: Effective concentration required to inhibit half of the maximum biological response (LPS-induced NO production)
LPS: Lipopolysaccharide
MTT: 3-(4, 5-dimethylthiazol-2-yl)-2, 5-diphenyl-tetrazolium bromide
NF-κB: Nuclear Factor-kappa-light-chain-enhancer of activated B
NO: Nitric Oxide
NOS2: Nitric oxide synthase 2
PGE2: Prostaglandin E2
RT-PCR: Reverse transcription polymerase chain reaction
STAT 3: Signal transducer and activator of transcription 3
TNF-α: Tumor necrosis factor alpha
