# Supplementary material for: Papaver nudicaule (Iceland poppy) alleviates lipopolysaccharide-induced inflammation through inactivating NF-κB and STAT3
Source: BMC Complement Altern Med. 2019 Apr 29;19:90. doi: 10.1186/s12906-019-2497-5 (PMC6489246; doi:10.1186/s12906-019-2497-5)
Supplement: Supplementary file 1 — Table S1. IC50 values of Papaver nudicaule cultivars. (DOCX 14 kb) [file 12906_2019_2497_MOESM1_ESM.docx]

**Supplementary Table 1**

IC_50_ values of *Papaver nudicaule* cultivars.

|  | Cultivation Period (days) | |
| --- | --- | --- |
|  | 60 | 90 |
|  | IC_50_ (μg/ml) | |
| NS | 3.62E ± 08 | 16,545 |
| NW | 2.13E ± 06 | 6.17 ± 06 |
| NY | 2.85E ± 07 | 2,059 |
| NP | 1.85E ± 07 | 2.52E ± 08 |
| NO | 110,350 | 4,731 |
