# Supplementary material for: Papaver nudicaule (Iceland poppy) alleviates lipopolysaccharide-induced inflammation through inactivating NF-κB and STAT3
Source: BMC Complement Altern Med. 2019 Apr 29;19:90. doi: 10.1186/s12906-019-2497-5 (PMC6489246; doi:10.1186/s12906-019-2497-5)
Supplement: Supplementary file 2 — Figure S1. Extracted ion chromatograms of (A) authentic standard of allocryptopine and (B) blank solution and allocryptopine from samples analyzed by LC-QTOF in the ethanol extracts of aerial parts of (C) NW (D) NO (E) NY (F) NS, and (G) NP at a cultivation period of 90 days. Supplementary Methods. The liquid chromatography-mass spectrometry system consisted of a Thermo Scientific Vanquish UHPLC system (Thermo Fisher Scientific, Sunnyvale, CA, USA) with an Acquity UPLC HSS T3 column (2.1 mm × 100 mm, 1.7 μm; Waters) and a Triple TOF 5600+ mass spectrometer system (Triple TOF MS; QTOF, Sciex, Foster City, CA, USA). Data acquisition and processing were carried out using Analyst TF 1.7, PeakVeiw 2.2 and MasterView software (Sciex, Foster City, CA, USA). (ZIP 185 kb) [file 12906_2019_2497_MOESM2_ESM.zip › supplementary material for AER3.pptx]

## Slide 1
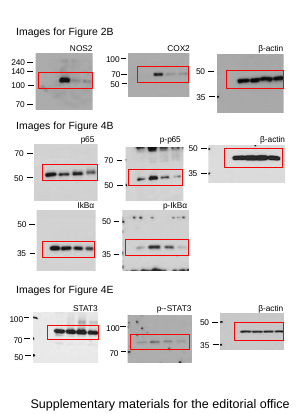

Images for Figure 2B
NOS2
240
140
100
70
β-actin
50
35
COX2
100
70
 50
Images for Figure 4B
p65
70
50
p-p65
70
50
β-actin
50
35
p-IkBα
50
35
IkBα
50
35
Images for Figure 4E
STAT3
100
70
50
p--STAT3
100
70
β-actin
50
35
Supplementary materials for the editorial office
