# Supplementary figures and images for: Papaver nudicaule (Iceland poppy) alleviates lipopolysaccharide-induced inflammation through inactivating NF-κB and STAT3
Source: BMC Complement Altern Med. 2019 Apr 29;19:90. doi: 10.1186/s12906-019-2497-5 (PMC6489246; doi:10.1186/s12906-019-2497-5)

## Slide 1
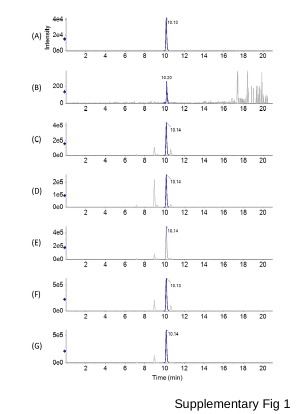

Supplementary Fig 1

Supplement: Supplementary file 2 — Figure S1. Extracted ion chromatograms of (A) authentic standard of allocryptopine and (B) blank solution and allocryptopine from samples analyzed by LC-QTOF in the ethanol extracts of aerial parts of (C) NW (D) NO (E) NY (F) NS, and (G) NP at a cultivation period of 90 days. Supplementary Methods. The liquid chromatography-mass spectrometry system consisted of a Thermo Scientific Vanquish UHPLC system (Thermo Fisher Scientific, Sunnyvale, CA, USA) with an Acquity UPLC HSS T3 column (2.1 mm × 100 mm, 1.7 μm; Waters) and a Triple TOF 5600+ mass spectrometer system (Triple TOF MS; QTOF, Sciex, Foster City, CA, USA). Data acquisition and processing were carried out using Analyst TF 1.7, PeakVeiw 2.2 and MasterView software (Sciex, Foster City, CA, USA). (ZIP 185 kb) [file 12906_2019_2497_MOESM2_ESM.zip › supplementary Fig 1R3.pptx]
